# Exploring DSSC Efficiency Enhancement: SQI-F and SQI-Cl Dyes with Iodolyte Electrolytes and CDCA Optimization

**DOI:** 10.3390/molecules28207129

**Published:** 2023-10-17

**Authors:** Sultan A. Al-horaibi, Abdel-Basit Al-Odayni, Mohammed ALSaeedy, Fares Hezam Al-Ostoot, Adel Al-Salihy, Abdulmajeed Alezzy, Arwa Al-Adhreai, Faizaa A. Saif, Salama A. Yaseen, Waseem Sharaf Saeed

**Affiliations:** 1 Department of Chemistry, Dr. Babasaheb Ambedkar Marathwada University, Aurangabad 431004, India; 2 Department of Restorative Dental Science, College of Dentistry, King Saud University, P.O. Box 60169, Riyadh 11545, Saudi Arabia; 3 Department of Chemistry, Maulana Azad of Arts, Science and Commerce, Aurangabad 431004, India; 4 DOS in Biochemistry, University of Mysore, Manasagangotri, Mysuru 570006, India; 5 MIIT Key Laboratory of Critical Materials Technology for New Energy Conversion and Storage, School of Chemistry and Chemical Engineering, Harbin Institute of Technology, Harbin 150001, China; 6 Chemistry Department, Dr. Rafiq Zakaria Centre for Higher Learning and Advance Research, Dr. BAM University, Aurangabad 431001, India; 7 Microwave Research Laboratory, Department of Physics, Dr. Babasaheb Ambedkar Marathwada University, Aurangabad 431004, India

**Keywords:** DSSCs, unsymmetrical (SQI-F-SQI-Cl) squaraine dyes, halogen-functionalized dyes, DFT analysis, DSSC efficiency

## Abstract

This investigation delves into the potential use of halogen bonding to enhance both the short-circuit current (*J_SC_*) and overall efficiency of dye-sensitized solar cells (DSSCs). Specifically, we synthesized two distinct dyes, SQI-F and SQI-Cl, and characterized them using FT-IR, ^1^HNMR, ^13^C NMR, and mass spectroscopy. These dyes are based on the concept of incorporating halogen atoms within unsymmetrical squaraine structures with a donor–acceptor–donor (D-A-D) configuration. This strategic design aims to achieve optimal performance within DSSCs. We conducted comprehensive assessments using DSSC devices and integrated these synthesized dyes with iodolyte electrolytes, denoted as Z-50 and Z-100. Further enhancements were achieved through the addition of CDCA. Remarkably, in the absence of CDCA, both SQI-F and SQI-Cl dyes displayed distinct photovoltaic characteristics. However, through sensitization with three equivalents of CDCA, a significant improvement in performance became evident. The peak of performance was reached with the SQI-F dye, sensitized with three equivalents of CDCA, and paired with iodolyte Z-100. This combination yielded an impressive DSSC device efficiency of 6.74%, an open-circuit voltage (*V_OC_*) of 0.694 V, and a current density (*J_SC_*) of 13.67 mA/cm^2^. This substantial improvement in performance can primarily be attributed to the presence of a σ-hole, which facilitates a robust interaction between the electrolyte and the dyes anchored on the TiO_2_ substrate. This interaction optimizes the critical dye regeneration process within the DSSCs, ultimately leading to the observed enhancement in efficiency.

## 1. Introduction

Dye-sensitized solar cells (DSSCs) stand out as a promising contender for developing colorful and transparent solar cells to be used in energy-harvesting decorative windows. However, achieving both high power conversion efficiency (PCE) and transparency in these solar cells requires skillful navigation of a delicate trade-off. DSSCs use organic dyes to transfer photons to electrons, exciting them to higher energy states [1,2]. Initially employing metal complex sensitizers, DSSCs have since evolved with advancements in p-type systems, metal–organic complexes, and metal-free organic dyes, expanding their potential [3,4].

Organic dye molecules offer advantages such as extensive absorption coefficients, cost-effectiveness, and long-term stability [5,6], making them a preferred choice. Various electron-donating groups have been explored in the design of metal-free sensitizers, showing promising performance. The strategic customization of hydrophobic alkyl groups within the sensitizer serves a dual purpose: passivating and safeguarding the surface, effectively restraining the recombination of electrons in TiO_2_ with oxidized electrolyte species [7,8,9,10,11]. This innovative design approach revolves around manipulating dyes to regulate the aggregation phenomenon on the TiO_2_ surface. It is widely acknowledged that key photovoltaic performance metrics such as *V_OC_* and *J_SC_* in DSSCs are significantly impacted by the kinetics of dye regeneration [12,13,14,15,16,17]. Consequently, the incorporation of supplementary elements to augment the dye regeneration process within the dye’s structure holds immense allure and indispensability, synergistically complementing the intrinsic electronic and steric characteristics of the sensitizers [18,19,20,21]. The remarkable directionality of halogen bonds has led to their versatile applications in both chemistry and chemical biology [22,23,24]. Their efficacy lies in the ability to efficiently regenerate oxidized dyes through non-covalent interactions involving halogen atoms in the dye and iodide ions present in the electrolyte. By incorporating polarizable atoms such as ^−^F or ^−^Cl into D-π-A-based sensitizers, in conjunction with I^−^/I_3_^−^-based electrolytes, the regeneration process is further enhanced [19,20].

A noteworthy advancement in regeneration kinetics and overall device performance has been achieved by facilitating a more favorable interaction between the dye and cobalt electrolyte through the introduction of a pyridyl unit in the dye’s molecular structure [25,26,27]. This highlights the importance of integrating functional moieties that facilitate the dye-regeneration process, whether via halogen bonding or exploiting the σ-hole in halogen atom-containing dyes [26]. The pursuit of improving the *J_SC_* and overall efficiency of DSSCs places significant emphasis on the incorporation of metal-free organic dyes [28,29,30,31,32,33]. This is mainly due to the scarcity of chromophores capable of effectively capturing photons in the far-red and near-infrared (NIR) regions of the solar spectrum [34,35,36,37,38,39,40,41,42]. Notably, unsymmetrical squaraine dyes (USQ) derived from polymethine chromophores exhibit an exceptionally high extinction coefficient in the far-red region, further underscoring their importance in DSSC applications [42,43]. Squaraine dyes (SQ) are promising candidates for serving as active molecules in DSSCs due to their high molar extinction coefficients. For example, Shivashimpi et al. achieved an energy conversion efficiency of approximately 5.03% when employing SQ dyes [44]. Our previous research has shown that we can readily adjust the absorption bands of SQ dyes through structural modifications, enabling them to align closely with the spectral response of sunlight, particularly in the NIR region [42].

This study investigates the potential of utilizing halogen bonding to enhance both the *J_SC_* (short-circuit current) and overall efficiency in DSSCs. We synthesized two distinct dyes, SQI-F and SQI-Cl, and characterized them using FT-IR, ^1^HNMR, ^13^C NMR, and mass spectroscopy techniques. These dyes incorporate halogen atoms within unsymmetrical squaraine structures, adopting a donor–acceptor–donor (D-A-D) configuration meticulously designed for optimal DSSC performance. Comprehensive assessments were conducted using DSSC devices, integrating these synthesized dyes with iodolyte electrolytes named Z-50 and Z-100. Further enhancements were achieved by introducing CDCA (chenodeoxycholic acid) into the DSSCs. Notably, in the absence of CDCA, both SQI-F and SQI-Cl dyes displayed distinct photovoltaic characteristics. However, upon sensitization with three equivalents of CDCA, significant performance improvement was observed. The highest level of performance was attained with the SQI-F dye, sensitized with three equivalents of CDCA, and paired with iodolyte Z-100. This combination yielded an impressive DSSC device efficiency of 6.74%, an open-circuit voltage (*V_OC_*) of 0.694 V, and a current density (*J_SC_*) of 13.67 mA/cm^2^. This enhancement can primarily be attributed to the presence of a σ-hole, facilitating a robust interaction between the electrolyte and the dyes anchored on the TiO_2_ substrate. This interaction optimizes the crucial dye regeneration process within the DSSCs, ultimately leading to the observed efficiency improvement.

## 2. Results and Discussion

### 2.1. Synthesis of Halogen-Functionalized SQI-F and SQI-Cl Dyes

**Figure 1:**
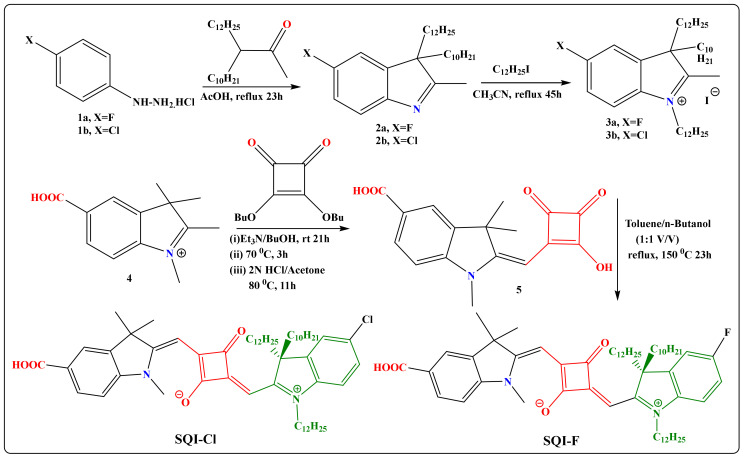
Synthesis pathway for the SQI-F and SQI-Cl dyes.

The synthesis of unsymmetrical squaraine dyes, specifically those containing halo-substituted atoms (SQI-F and SQI-Cl), primarily involves the condensation of specialized indolium salts with Dibutyl squarate. This synthetic pathway begins with the halo-substituted 4-hydrazinohydrochloride derivatives, labeled as compounds **1a** and **1b**, reacting with 3-Methyl-2-butanone, resulting in the formation of halogen-substituted indoline compounds, referred to as **2a** and **2b**. These freshly synthesized indoline derivatives (**2a** and **2b**) then undergo a reaction with 1-iodododecane, yielding the corresponding indolium salts, specifically designated as compounds **3a** and **3b**. In the subsequent phase, indolium salts **3a** and **3b** react with a precursor known as the semi-squaraine dye (5) under azeotropic conditions, which are carefully monitored to ensure effective water extraction. This process ultimately leads to the formation of unsymmetrical squaraine dyes containing halogen groups, namely SQI-F and SQI-Cl (as illustrated in Figure 1). The synthesis process shows favorable yields, with percentages ranging from 43% to 56%. The resulting dye compounds appear as deep blue solids and demonstrate solubility in a variety of solvents, including ethanol, DMSO, methanol, and chloroform. Characterization of these dyes is achieved through techniques such as FT-IR, ^1^HNMR, ^13^C-NMR, and mass spectrometry.

### 2.2. Evaluation of Photophysical and Electrochemical Attributes

**Figure 2:**
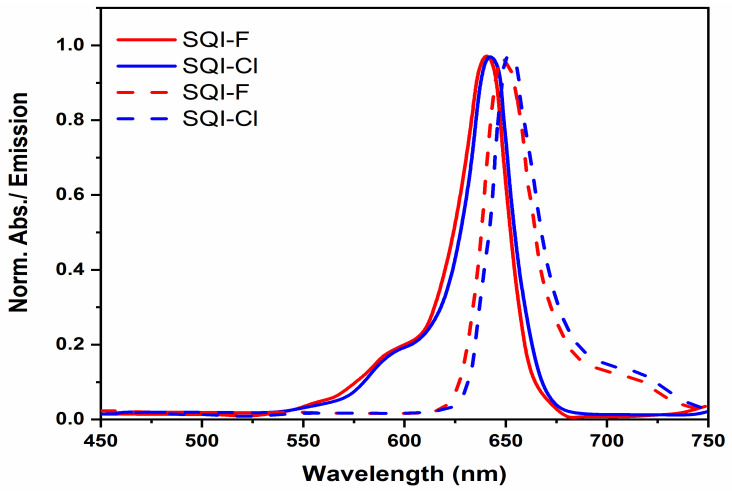
The normalized UV-visible and emission spectra of SQI-F and SQI-Cl in an EtOH solution.

The SQI-F and SQI-Cl dyes underwent an exhaustive examination, exploring both their photophysical and electrochemical properties in an ethanol solution. Within the framework of unsymmetrical squaraine dyes relying on indoline donors, the UV-visible absorption spectrum of these solutions presents a clear and prominent peak, boasting an absorptivity exceeding 10^5^ M^−1^ cm^−1^, positioned around the 641 and 643 nm regions. This characteristic peak arises from the π-π* electronic transition, accompanied by a vibronic side peak, observed as a higher-energy shoulder at 593 nm. Figure 2 visually illustrates the normalized absorption and emission spectra for both SQI-F and SQI-Cl. An observable redshift is apparent in the absorption bands of the SQI-F and SQI-Cl dyes, with a peak wavelength (*λ*_max_) at 641 nm in contrast to SQI-Cl’s *λ*_max_ at 643 nm. Similar patterns can be observed in the emission spectra, with SQI-F emitting at 651 nm relative to SQI-Cl’s emission at 653 nm. The molar extinction coefficients for both SQI-F and SQI-Cl dyes fall within the range of (2.1 and 2.5) × 10^5^ M^−1^ cm^−1^.

**Figure 3:**
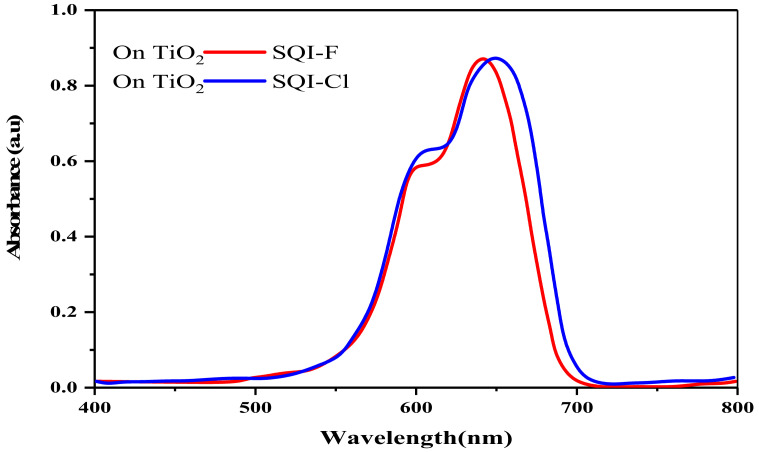
The UV-visible absorption spectra of SQI-F and SQI-Cl dyes as they interact with the TiO_2_ electrode.

UV-visible characterizations were performed on TiO_2_ surfaces to evaluate the aggregation tendencies of SQI-F and SQI-Cl dyes, as well as their light harvesting capabilities. Electrode immersion durations of 10 min and 15 h were employed, utilizing a 0.1 mM dye solution in ethanol (as shown in Figure 3 and Table 1). Notably, all SQI-F and SQI-Cl dyes displayed redshifted charge transfer (π-π*) peaks, with wavelengths of 642 and 651 nm, respectively. Upon the introduction of the optically transparent co-adsorbent CDCA, a significant competitive adsorption of CDCA onto the metal–oxide interface was observed.

**Figure 4:**
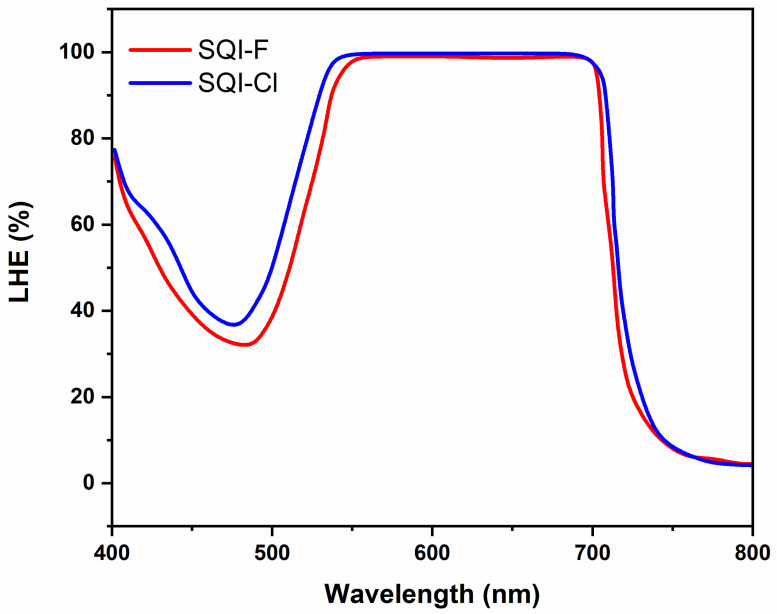
The LHE for SQI-F and SQI-Cl dyes on the TiO_2_ electrode is denoted as LHE = 1–10^−A^.

Light-harvesting efficiency (LHE) plays as a pivotal role in the field of DSSCs, providing insights into the ability of sensitizers to effectively capture photons across distinct wavelength intervals corresponding to specific dye molecules. The LHE characteristics associated with SQI-F and SQI-Cl dyes are prominently illustrated in Figure 4 and elaborated in detail in Table 1. Notably, these dyes, upon integration onto mesoporous TiO_2_ thin films, exhibit a well-defined and significant spectral absorbance spectrum across the 490–750 nm wavelength range. Adding to this characterization is the evident broadening of the spectral range, with an incremental Δ*λ* of 195 and 213 nm, respectively, for SQI-F and SQI-Cl, while maintaining the LHE at the 60% threshold. This phenomenon underscores the substantial degree of intermolecular interactions among the dye molecules situated on the surface of the TiO_2_ substrate.

In Table 1, we examine the photophysical and electrochemical characteristics of SQI-F and SQI-Cl, evaluating various critical parameters. The emission wavelength (*λ*_em_) for both dyes was determined to be 643 nm and 653 nm, respectively. When interacting with TiO_2_, the *λ*_max_ TiO_2_ was recorded at 642 nm and 651 nm. Notably, a noteworthy observation was the wavelength shift of LHE at 60%, measuring 195 nm for SQI-F and 213 nm for SQI-Cl. The oxidation potentials (*E*_oxd_) in volts versus the normal hydrogen electrode (NHE) were 0.81 V and 0.83 V, indicating a slight increase in the oxidation tendency for SQI-Cl. The band gap energies (*E*_g_) were quite similar, with values of 1.92 eV and 1.93 eV. The energy levels of the HOMOs were found to be −6.01 eV and −6.03 eV, while those of the LUMOs were at −4.09 eV and −4.1 eV for SQI-F and SQI-Cl, respectively. These findings provide valuable insights into the electronic structure and photophysical behavior of the two dyes, setting the stage for further exploration of their potential applications in DSSCs. The data demonstrate how halogen substitutions can subtly alter the photophysical properties of the dyes. The differences in absorption wavelengths, molar absorptivity, and oxidation potentials might influence the performance of these dyes in DSSCs. The analysis suggests that careful tuning of the halogen substituent could lead to improved light harvesting efficiency and optimized electronic properties for DSSC applications.

**Figure 5:**
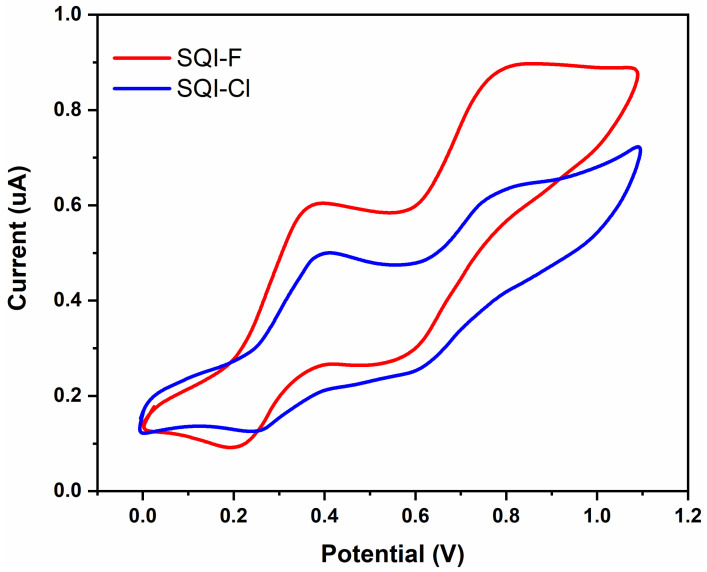
Cyclic voltammograms for both SQI-F and SQI-Cl.

Cyclic voltammetry (CV) analysis was conducted using a medium consisting of a 0.1 M solution of TBAPF6 in DMSO, with dyes SQI-F and SQI-Cl present at concentrations of 0.001 M. A scan rate of 50 mV/s was applied, covering a potential scan range from −0.1 to 1.2 V (as shown in Figure 5). The comprehensive characterization of the photophysical and electrochemical properties of the dyes is summarized in Table 1. Graphical representation of the cyclic voltammetry (CV) data allowed for the determination of oxidation potentials (E_oxd_) for SQI-F and SQI-Cl, which were recorded at 0.81 V and 0.83 V, respectively. These values correspond to absolute energy levels of −6.01 and −6.03 eV. The calculation of the highest occupied molecular orbital (HOMO) energy levels was carried out using Equation (1). This process also involved establishing a correlation between ferrocene’s HOMO energy level relative to vacuum, as detailed in Reference [2].

(1) $$E_{HOMO}=-\left(E_{OX}^{Onest}-E_{\frac{FC}{FC}+}^{Onest}\right)-4.8$$

The calculation of the LUMO energies was obtained using the following mathematical expression:

*E_LUMO_* = *E_HOMO_* − *E_g_* (2)

Moreover, the recorded *E*_ox_ values (HOMO) for SQI-F and SQI-Cl displayed substantial positive deviations when compared to the redox potential of the iodide/triiodide complex used as the electrolyte. This difference highlights their effectiveness as efficient electron donors. In contrast, the determined E_red_ values (indicative of the lowest unoccupied molecular orbital, LUMO) for SQI-F and SQI-Cl exhibited appropriately negative characteristics, thus confirming the feasibility and viability of electron injection into the CB of TiO_2._

### 2.3. Calculations Using Density Functional Theory (DFT)

In our current research framework, we employed computational techniques based on density functional theory (DFT) to explore the molecular orbital structures in ethanol (EtOH). The effective incorporation of EtOH’s solvation dynamics was achieved using the conductor-like polarizable continuum model (C-PCM). All of these computational processes were conducted using the Gaussian 09 platform, and visual representations of the data were generated using GaussView software (version 5.0.8) [45]. Subsequent analyses have shown that the fundamental electronic structures, particularly the HOMOs and LUMOs, remain largely unaffected by the introduction of halogen atoms. In this context, the rapid movement of electrons from the dye to the conductive continuum of TiO_2_ when exposed to light is of utmost importance. At the same time, ensuring the maintenance of charge separation at the interface following this electronic transfer is crucial. To enhance this mechanism, it is necessary for the HOMO to be positioned away from TiO_2_, acting as a barrier to retrograde electron pathways. Additionally, aligning the LUMO in close proximity to the TiO_2_ layer enhances the efficiency of electron transfer from the HOMO to the LUMO [46,47].

**Figure 6:**
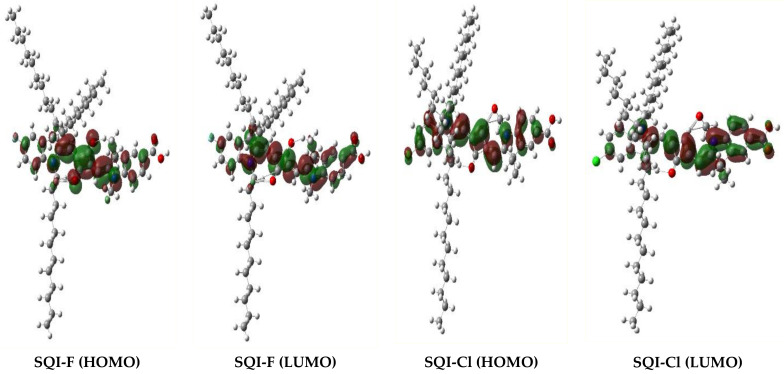
Frontier molecular orbitals of the HOMO and LUMO for the SQI-F and SQI-Cl sensitizers.

In addition to refining structural details within the unsymmetrical squaraine-infused indoline, theoretical computations were initiated to determine the energetic levels of the HOMOs and LUMOs for SQI-F and SQI-Cl. These findings are illustrated in Figure 6 and summarized in Table 1. The primary motivation behind incorporating unsymmetrical squaraine dyes in DSSCs is to enhance the efficiency of converting light energy into electricity. Notably, unsymmetrical squaraine sensitizers, SQI-F and SQI-Cl, exhibit improved performance compared to their conventional counterparts due to their higher molar extinction coefficient, which enhances their light-absorbing capabilities. Their increased stability, when compared to typical dyes, further underscores their suitability for DSSC applications. Regarding their electronic configurations, SQI-F and SQI-Cl dyes exhibit HOMO distributions primarily over the polymethine backbone, with reduced electron densities at the indoline donors. In contrast, the LUMO is primarily distributed over the carboxylic acid-endowed donor sites, aligning with the polymethine structure. This design facilitates streamlined charge injection following photo-excitation. When interacting with the TiO_2_ layer, an enhanced charge density distribution across the LUMOs is expected to enhance stability within the dye sensitizer. Additionally, the presence of carboxylic acid (-COOH) moieties provides favorable sites for electron transfer from SQI-F and SQI-Cl dyes to the TiO_2_ surface. To establish a seamless electronic connection between the photo-excited dye molecule and the extensive D-orbitals of the wide-band gap semiconductor, it is imperative to have sufficient electron density at the dye’s anchoring group. This arrangement facilitates unimpeded and rapid electron transfer from the dye’s LUMO to the receptive conduction band (CB) of TiO_2_. This hypothesis is supported by the electron density dispersion within the LUMOs of SQI-F and SQI-Cl, as depicted in Figure 6. This dispersion not only demonstrates intrinsic charge transfer and coplanarity with the primary molecular structure but also directs a significant electron density to the anchoring group. This arrangement creates an optimal force for propelling electrons into the CB of TiO_2_, highlighting the suitability of SQI-F and SQI-Cl for DSSC applications. In terms of energy, the HOMOs for SQI-F and SQI-Cl were determined to be −5.14 and −5.16 eV, respectively. These values are lower than the redox potential of I^−^_3_/I^−^ (−4.8 eV), which enhances the feasibility of the dye regeneration process. The HOMO values were determined to be −3.13 eV for SQI-F and −3.17 eV for SQI-Cl, as shown in Table 1. Furthermore, the LUMOs of these dyes surpass the energy level of the TiO_2_ (CB), enabling efficient electron transfer from the dyes to the TiO_2_ semiconductor. Overall, these explanations confirm that SQI-F and SQI-Cl exhibit a favorable alignment of HOMO and LUMO energy levels with the TiO_2_ conduction band and the redox potential of I^−^_3_/I^−^, meeting the necessary conditions for effective DSSCs.

**Figure 7:**
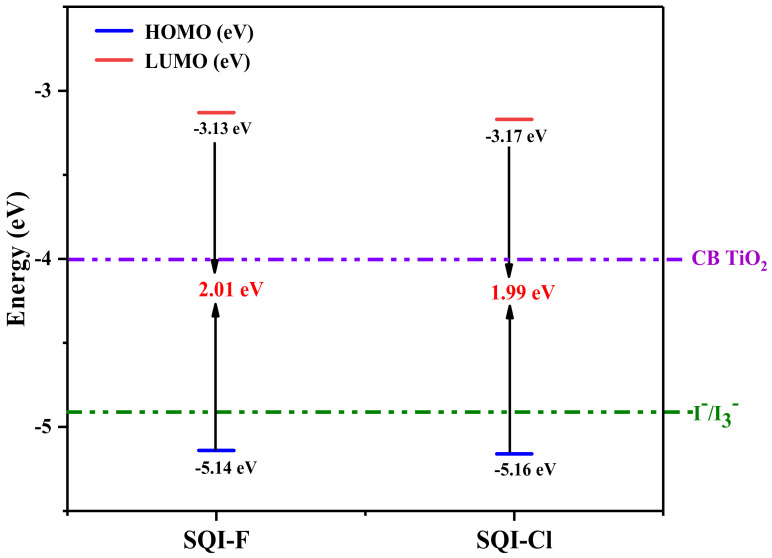
Energy band gap (*E*_g_) diagram for SQI-F and SQI-Cl computed using the B3LYP/6-311++G (p, d) basis set.

The effective functioning of DSSCs relies heavily on the precise alignment of energy levels among its core components: TiO_2_, SQI-F and SQI-Cl dyes, and the I^−^/I_3_^−^ redox pair. When photons interact with dye molecules, they transition to their LUMO state, facilitating the movement of electrons to the conduction band (CB) of the TiO_2_ semiconductor. To achieve optimal electron transfer, it is essential to maintain a continuous energy pathway between the dye states, TiO_2_, and I^−^/I_3_^−^. Therefore, aligning the energy levels of the sensitizer with both the wide-band gap semiconductor (*E_g_*) and I^−^/I_3_^−^ is crucial in determining its suitability for DSSC applications. It is worth noting that TiO_2_ and I^−^/I_3_^−^ consistently play significant roles in shaping the *E_g_* diagrams for DSSCs sensitizers, given their respective positions as the wide-band gap semiconductor and the redox pair I^−^/I_3_^−^. Based on existing research, the redox potential of I^−^/I_3_^−^ and the CB energy level of TiO_2_ are approximately −4.00 eV and −4.90 eV, respectively. As depicted in Figure 7, the LUMO energies of all sensitizers exceed the CB energy thresholds of TiO_2_, ensuring efficient electron injection after photon interaction. Furthermore, the HOMO energy levels of the dyes, compared to the redox potential of I^−^/I_3_^−^, support the timely regeneration of oxidized dyes following electron flow.

### 2.4. Characterization of Photovoltaic (SQI-F and SQI-Cl) Properties

**Figure 8:**
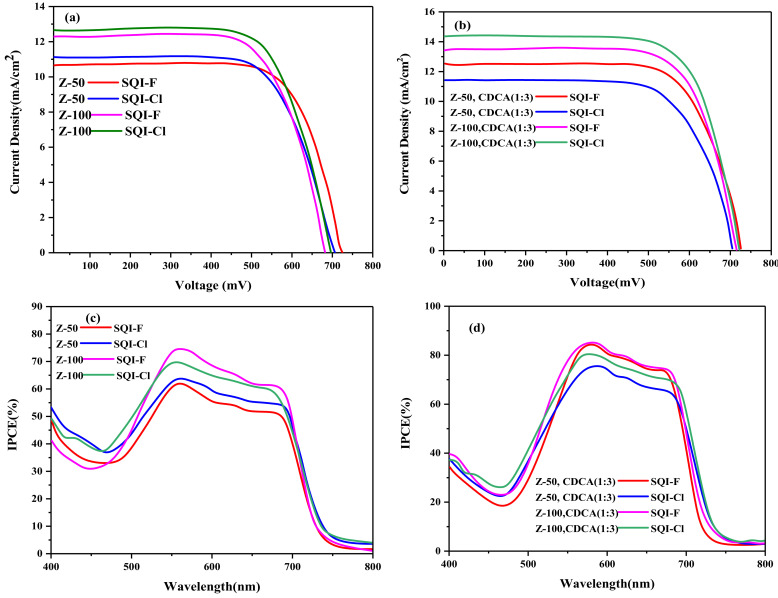
*I–V* curves (**a**,**b**) and *IPCE* curves (**c**,**d**) for SQI-F and SQI-Cl, illustrating variations with and without the conditions Z-50 and Z-100.

The detailed investigation into the photovoltaic characteristics of halogen-infused dyes (SQI-F and SQI-Cl) was meticulously conducted by utilizing a pair of iodolyte electrolytes (I^−^/I_3_^−^) and has been systematically optimized with varying concentrations of CDCA. Unsymmetrical squaraine sensitizers (USQ) are emerging as promising entities in the field of NIR-DSSCs, showcasing distinguished photovoltaic efficiency. These unique molecules display vigorous absorption properties in the NIR region, enabling them to capture photons that are typically beyond the reach of standard DSSCs. The specific squaraines SQI-F and SQI-Cl enhance this effect with a high molar extinction coefficient, further boosting the light-harvesting efficiency of the cells. Despite these remarkable attributes, the employment of USQ in NIR-DSSCs is not without challenges. Pursuing optimal performance demands the resolution of specific hurdles, such as the discovery of suitable electron transport materials and the enhancement of the solar cells’ stability. As a result, sustained research and innovative efforts are imperative to unlock the full capabilities of USQ within the context of NIR-DSSCs technology. DSSC devices were carefully constructed, utilizing two separate concentrations of iodolyte. Specifically, the I_3_^−^ concentrations were selected with great precision, with them being 50 mM for the Z-50 variant and 100 mM for the Z-100 variant. Figure 8 and Table 2 delineate the photovoltaic characteristics, specifically (*V*_OC_, *J*_SC_, *ff,* and *η*). Utilizing iodolyte Z-50 without CDCA in the synthesis of the DSSC device resulted in suboptimal *J*_SC_ and efficiency for the SQI-F dye in comparison to its hydrogen-containing counterpart, SQI-Cl. The adoption of iodolyte Z-100 as the electrolyte increased *J*_SC_ while simultaneously reducing *V*_OC_ for all dyes absent of CDCA. As a result, the highest efficiency recorded was 6.01% for SQI-F (*V*_OC_ of 0.693 V; *J*_SC_ of 12.4 mA/cm^2^) in contrast to 5.84% for SQI-Cl (*V*_OC_ of 0.683 V; *J*_SC_ of 12.22 mA/cm^2^).

The fabrication of devices incorporating three equivalents of CDCA resulted in an increase in *V_OC_*, *J*_SC_, and efficiency for both SQI-F and SQI-Cl dyes in the iodolyte solutions of Z-50 and Z-100, in comparison to those without CDCA sensitization. In these electrolyte configurations, the SQI-F dye, when not combined with CDCA, exhibited superiority in the DSSC device efficiency over the SQI-Cl dye. The highest DSSC device efficiency was achieved at 6.74% (with a *V*_OC_ of 0.694 V and *J*_SC_ of 13.67 mA/cm^2^) using the SQI-F dye in the iodolyte Z-100 formulation, sensitized with three equivalents of CDCA. In contrast, the peak efficiency for SQI-Cl reached 5.64% for SQI-Cl (with a *V*_OC_ of 0.680 V and *J*_SC_ of 12.09 mA/cm^2^). Both SQI-F and SQI-Cl dyes exhibited exceptional IPCE responses across the visible and NIR spectra. The improvement in IPCE for these dyes in the Z-100 iodolyte matrix contributed to the increased *J_SC_* compared to the Z-50 variant. Furthermore, the higher concentrations of the electrolyte led to the strengthening of halogen bonding between the oxidized forms of SQI-F and SQI-Cl dyes and the iodide ions on the TiO_2_ surface. This interaction accelerated the dye regeneration process, consequently enhancing the *J_SC_* of the structures. The reduced performance of SQI-Cl in DSSCs compared to SQI-F can be attributed to the electronic influence of the chloride atom on the indoline moiety. Finally, Figure 8 visually presents *V_OC_* and *J_SC_* in iodolyte solutions (Z-50 and Z-100) for SQI-F and SQI-Cl dyes, both in the presence and absence of CDCA. The significant increase in photocurrent generation with Z-100, when compared to Z-50, highlights the interactions between the electrolyte and the oxidized halogen atom in the SQI-F dye, as opposed to SQI-Cl. The data suggest that both CDCA and the type of iodolyte have an impact on photovoltaic parameters, with SQI-F dye demonstrating better overall performance, especially in the Z-100 electrolyte with 0.3 mM CDCA.

The comparative analysis of photo-electrochemical characteristics between SQI-F and SQI-Cl, with and without CDCA, in Z-50 and Z-100 electrolytes, along with SQIND-1 and SQ-A, reveals important distinctions. SQI-F, in general, outperforms SQI-Cl, exhibiting the highest *Jsc* (short-circuit current density) at 12.4 mA/cm^2^ without CDCA in Z-100, surpassing all other configurations (Table 2). CDCA’s presence leads to enhanced *Jsc* for both dyes in various electrolytes, with SQI-F in Z-100 still performing notably well. Meanwhile, the *FF* (fill factor) remains relatively stable across configurations, with it hovering between 0.72 and 0.75. SQIND-1 demonstrates comparatively lower *Jsc*, *Voc*, *FF*, and *η* (energy conversion efficiency) values, indicating its inferior photoelectrochemical performance. SQ-A, although better than SQIND-1, still falls short of SQI-F and SQI-Cl. These findings underscore the significance of selecting appropriate materials and additives for photoelectrochemical systems for DSSC applications, shedding light on performance variations that can guide optimization strategies.

### 2.5. Insights from Electrochemical Impedance Spectroscopy (EIS)

**Figure 9:**
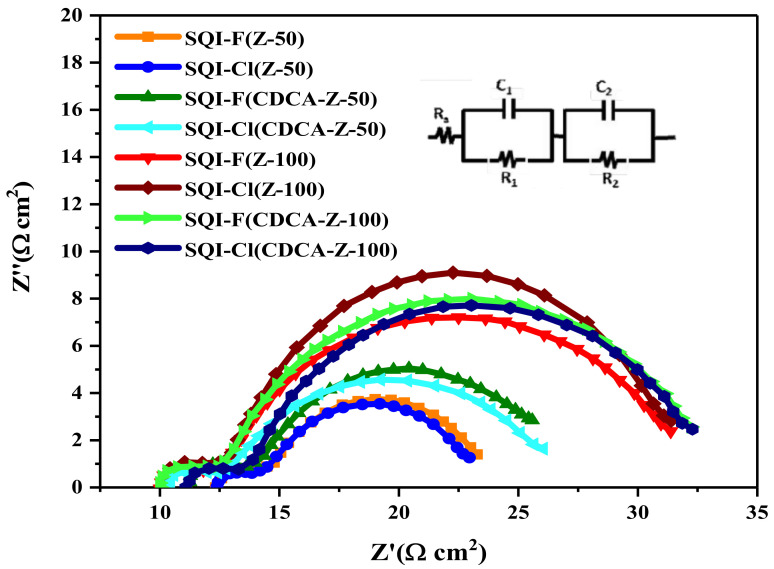
Nyquist plots for SQI-F and SQI-Cl dyes when combined with CDCA, using the iodolyte electrolytes Z-50 and Z-150.

The EIS technique was used to gain a deeper understanding of key parameters including the charge recombination resistance (R_ct_), chemical capacitance (C_μ_), and the lifetime of electrons (τ) at the interface of the dye-TiO_2_ and the electrolyte. This study specifically focused on the SQI-F and SQI-Cl dyes, which contain halogen, and was conducted under non-illuminated conditions while varying applied potentials. Two different iodolyte variants, Z-50 and Z-100, were employed for this investigation. A comprehensive presentation of the EIS results, especially when CDCA is incorporated, can be found in Figure 9 and Table 3. Given the similar steric characteristics of SQI-F and SQI-Cl dyes, there were no significant differences in the values of R_ct_, C_μ_, and τ, regardless of the specific iodolyte (Z-50 or Z-100) used during the device fabrication in combination with CDCA. Remarkably, the highest values of 5.52 Ω cm^2^ (R_ct_), 0.403 mF (C_μ_), and 2.22 ms (τ) were observed for the SQI-Cl dye when used in combination with the Z-100 iodolyte and supplemented with CDCA. This suggests that it may exhibit enhanced efficiency in device performance compared to the SQI-F dye. When we observed that the higher Rct values obtained from EIS do not perfectly correspond to the lower *V_OC_* values in DSSC devices when using the Z-100 electrolyte, the introduction of a different functional group into the fundamental structure of the dye might influence its inherent characteristics, such as the dipole moment. These modifications could, in turn, have an impact on the positioning of the TiO_2_ conduction band, leading to variations in the *V_OC_* measurements of the devices, even in the presence of a noticeable increase in Rct, as indicated by the EIS data. The observed changes in solution resistance values, as outlined in Table 3, provide valuable insights into the complex interaction between specific dye compositions and the presence of chenodeoxycholic acid (CDCA) in the solution. Notably, for SQI-F, the addition of CDCA in a 1:3 ratio results in a noticeable increase in solution resistance, indicating altered electrochemical characteristics. Conversely, in the case of SQI-Cl, a subtle decrease in resistance is observed upon the inclusion of CDCA. These shifts in solution resistance highlight the nuanced impact of CDCA on the electrical behavior of the respective dye solutions, leading to varied impedance properties. This variation could potentially influence their suitability and performance in different electrochemical applications.

## 3. Materials and Methods

Experimental Methods: The synthesis of SQI-F and SQI-Cl dyes involved the acquisition of all necessary chemical reagents from Sigma-Aldrich (Taufkirchen, Germany), followed by standard procedures being followed for solvent drying. To elucidate the molecular structures, advanced NMR spectroscopy techniques were employed, recording precise ^1^HNMR and ^13^C-NMR spectra on Bruker NMR spectrometers operating at varying frequencies of 400, and 500 MHz, utilizing CDCl_3_ as the solvent. Additionally, the ABSciex 5800 MALDI TOF mass spectrometer was utilized for MALDI-TOF-MS analysis. To investigate the absorption spectra of the SQI-F and SQI-Cl dyes, experiments were conducted at room temperature using a high-performance SPECORD^®^ 210/PLUS, Analytikjena UV-Visible spectrophotometer. The light-harvesting efficiency (LHE) was determined by submerging a 6 μm thick TiO_2_-coated transparent glass slide in a 0.1 mM solution of SQI-F to SQI-I dyes for 14 h under controlled ambient conditions. Following this, the absorption spectra were precisely obtained using the SPECORD^®^ 210/PLUS, Analytikjena UV-visible spectrophotometer, from which the LHE was calculated (LHE = 1–10^(−A)^). Fluorescence and lifetime measurements of the SQI-F and SQI-Cl dyes were conducted using the state-of-the-art Edinburgh spectrofluorometer FS5, with precise control of the experimental environment at room temperature. For investigating the cyclic voltammetry (CV) behavior of the SQI-F and SQI-Cl dyes, the advanced Biologic SP300 potentiostat was utilized in anhydrous DMSO solvent at 25 °C, under an inert atmosphere. Moreover, the optical band gaps (E_g_) of the SQI-F and SQI-Cl dyes were computed as 1240/*λ*, where *λ* represented the point of intersection between the absorption and fluorescence spectra. For electrochemical impedance measurements (EIS), a state-of-the-art BioLogic SP300 potentiostat, outfitted with a frequency response analyzer, was utilized for the experiments. EIS investigations were carried out under dark conditions, spanning various set potentials and frequency ranges from 3 to 10 MHz. A sinusoidal amplitude of 10 mV was maintained to guarantee precise and detailed analyses. The solar efficiency of the fabricated DSSC devices using SQI-F and SQI-Cl dyes was assessed using a solar simulator from Dayton Instruments, USA, coupled with a Keithley 2400 source meter. The incident photon to current efficiency (IPCE) was rigorously analyzed with the Newport QE measurement apparatus, shedding light on the device’s capacity to translate incoming photons into an electrical current. Computational evaluations of the SQI-F and SQI-Cl dyes were conducted using the Gaussian 09 software package. Ground-state geometry optimization was performed with the DFT approach, utilizing the robust B3LYP/6-311++G(p, d) basis set. These structures were elucidated using Becke’s triparametric exchange functional and the Lee–Yang–Parr gradient-refined correlation functional, both integrated into the comprehensive Gaussian 09 computational suite.

### 3.1. Synthesis and Characterization

#### 3.1.1. Synthesis of Compound (**2a**)

In a round-bottom flask, 1 g (6.15 mmol) of Floro-phenylhydrazine hydrochloride (**1a**) was combined with 2.92 g (7.98 mmol) of 3-decylpentadecan-2-one. The compounds were dissolved in 10 mL of acetic acid (AcOH). The resulting mixture was then heated under reflux conditions under a nitrogen atmosphere for 23 h. After allowing the mixture to cool, the solvents were removed under reduced pressure. The residual material was then purified using column chromatography (with a 60–120 mesh size) with a mixture of petroleum ether and ethyl acetate (EtOAc) in a ratio of 10:0.5 (*V*/*V*) as eluents, resulting in a yield of 61%. ^1^H NMR-CDCl_3_-400 MHz: δ 0.79 (t, 2CH_3_, 6H), 0.91–0.94 (m, 4CH_2_, 8H), 1.22–1.33 (m, 14CH_2_, 28 H), 1.74–1.83 (m, 2CH_2_, 4H), 2.21 (s, CH_3_, 3H), 7.34 (d, Ar-H, 1H, *J* = 8.1 Hz), 7.22 (dd, Ar-H, 1H, *J* = 4 Hz), 7.16 (d, Ar-H, 1H *J* = 8.1 Hz). ^13^CNMR-CDCl_3_: δ 14.3, 16.8, 23.5, 24.4, 28.4, 30.1, 30.4, 30.6, 32.7, 33.3, 37.8, 46.69, 64.1, 110.2, 115.0, 121.0, 145.3, 152.0, 162.1, 187.3. MALDI-TOF mass spectrum confirmed the product’s structure with [M]^+^ at *m*/*z* 457.387.

#### 3.1.2. Synthesis of Compound (**2b**)

In a round-bottom flask, 1 g (5.58 mmol) of Chloro-phenylhydrazine hydrochloride (1b) was combined with 2.66 g (7.26 mmol) of 3-decylpentadecan-2-one. The compounds were dissolved in 10 mL of acetic acid (AcOH). The resulting mixture was then heated under reflux conditions under a nitrogen atmosphere for 23 h. After allowing the mixture to cool, the solvents were removed under reduced pressure. The residual material was then purified using column chromatography (with a 60–120 mesh size) with a mixture of petroleum ether and ethyl acetate (EtOAc) in a ratio of 10:0.5 (*V*/*V*) as eluents, resulting in a yield of 52%.^1^H NMR-CDCl_3_-400 MHz: δ 0.78 (t, 2CH_3_, 6H), 0.89–0.92 (m, 4CH_2_, 8H), 1.20–1.34 (m, 14CH_2_, 28 H), 1.71–1.79 (m, 2CH_2_, 4H), 2.22 (s, CH_3_, 3H), 7.32 (d, Ar-H, 1H, *J* = 8.1 Hz), 7.21 (dd, Ar-H, 1H, *J* = 4 Hz), 7.16 (d, Ar-H, 1H *J* = 8.1 Hz). ^13^CNMR-CDCl_3_: δ 14.2, 16.18, 22.7, 23.8, 28.6, 30.3, 30.5, 31.0, 32.5, 34.3, 37.9, 46.63, 64.2, 111.2, 116.0, 120.0, 146.3, 152.1, 162.2, 187.2. MALDI-TOF mass spectrum confirmed the product’s structure with [M]^+^ at *m*/*z* 457.385.

### 3.2. Synthesis of Halogen-Substituted Iodolium Salts (Compounds ***3a*** and ***3b***)

In a 50 mL round-bottom flask, 1-iodododecane (1.3 equiv.) was combined with 3-decyl-3-dodecyl-5-halo-2-methyl-3H-indole in a 1:1 ratio. The mixture was dissolved in 10 mL of CH_3_CN and then heated and refluxed under a nitrogen atmosphere for 45 h. Afterward, it was cooled to RT, and the solvent was removed by evaporation under reduced pressure. The remaining product material underwent five washes, each with 3 mL of n-pentane, resulting in the compound being a thick liquid with a brown–red color.

#### 3.2.1. Synthesis of Compound (**3a**)

Compound (**3a**) was obtained from an initial mixture of **2a** (1 g, 2.184 mmol) and 1-iodododecane (0.84 g, 2.84 mmol) in 10 mL of CH_3_CN. The mixture was heated and refluxed under a nitrogen atmosphere for 45 h, resulting in the compound being a thick liquid with a brown–red color, yielding 68%.^1^HNMR-CDCl_3_-400 MHz: δ 0.81–0.78 (m, 4CH_3_, 12H), 1.13–1.22 (m, 27CH_2_, 54H), 1.72–1.79 (m, CH_2_, 2H), 2.09–2.11 (m, 2CH_2_, 4H), 4.73 (t, CH_2_, 2H, *J* = 7.19 Hz), 7.17 (m, 1H, Ar-H), 7.23 (m, 1H, Ar-H), 7.76 (m, 1H, Ar-H). ^13^CNMR-CDCl_3_: δ 14.2, 16.8, 18.4, 23.5, 25.09, 27.8, 29.4, 30.2, 30.4, 31.3, 32.7, 34.4, 38.1, 51.8, 64.7, 112.3, 118.0, 118.6, 121.0, 139.1, 142.2, 161.9, 196.3.

#### 3.2.2. Synthesis of Compound (**3b**)

Compound (**3b**) was obtained from an initial mixture of **2b** (1 g, 2.11 mmol) and 1-iodododecane (0.812 g, 2.74 mmol) in 10 mL of CH_3_CN. The mixture was heated and refluxed under a nitrogen atmosphere for 45 h, resulting in the compound being a thick liquid with a brown–red color, yielding 74%.^1^HNMR-CDCl_3_-400 MHz: δ 0.81–0.86 (m, 4CH_3_, 12H), 1.12–1.27 (m, 26CH_2_, 52H), 1.81–1.85 (m, 2CH_2_, 4H), 2.09–2.11 (m, 2CH_2_, 4H), 4.88 (t, CH_2_, 2H, *J* = 7.19 Hz), 7.27 (m, 1H, Ar-H), 7.53 (m, 1H, Ar-H), 7.79 (m, 1H, Ar-H). ^13^CNMR-CDCl_3_: δ 14.1, 17.0, 18.5, 23.6, 24.4, 30.2, 30.4, 31.6, 32.8, 34.5, 46.5, 52.0, 64.3, 64.8, 117.8, 125.0, 131.1, 137.7, 141.5, 141.7, 196.8.

### 3.3. Synthesis of Semi-Squaraine Dye (***5***)

To synthesize compound (**5**), a mixture of compound (**4**) (1 g, 4.6 mmol) and 3,4-dibutoxycyclobut-3-ene-1,2-dione (1.04 g, 4.6 mmol) was dissolved in 10 mL of 1-butanol. Triethylamine (NEt3) (0.56 g, 5.52 mmol, 1.2 equiv.) was gradually added to the solution. The mixture was stirred for 15 h, followed by heating to 70 °C for 3 h and cooling to RT. The resulting mixture was purified by column chromatography (60–120 mesh) using a petroleum ether/EtOAc solvent mixture in a 10:0.5 (*V*/*V*) ratio, resulting the compound being a bright yellow solid. This product was dissolved in 15 mL of acetone, and 6 mL of 2N-HCl was added. The mixture was refluxed for an additional 8 h and then cooled, and the solvent was removed using a rotary evaporator, resulting in the compound (**5**) being a deep yellow solid with a yield of 92%.^1^HNMR-DMSO-d_6_, 500 MHz: δ 1.56 (s, 2CH_3_, 6H), 3.36 (s, CH_3_, 3H), 5.51 (s, =CH-, 1H), 7.02 (d, 1H, Ar-H, *J* = 8 Hz), 7.13 (d, 1H, Ar-H, *J* = 8 Hz), 7.38 (s, 1H, Ar-H, *J* = 10 Hz). ^13^CNMR-DMSO-d_6_: δ 26.7, 29.7, 46.9, 81.6, 108.6, 121.8, 121.9, 127.7, 140.1, 143.2, 166.9, 173.8, 190.2, 191.8. HRMS (*m*/*z*): [M + H]^+^ calculated for C_17_H_16_NO_5_: 314.1023, found, 314.1014.

### 3.4. Synthesis of Unsymmetrical Squaraine Dyes: SQI-F and SQI-Cl

For the synthesis of the unsymmetrical dyes, a mixture of compounds (**3a** and **3b**) and compound **5** was dissolved in a 50 mL round-bottom flask in a mixture of n-butanol/toluene (20 mL, 1:1 *V*/*V*). The mixture was heated and refluxed under an inert atmosphere for 22 h using a Dean–Stark apparatus. After cooling to room temperature, the solvent was removed. The resultant crude product was purified by column chromatography (60–120 mesh) using a mixture of DCM/MeOH in a 10:0.1 (*V*/*V*) ratio to obtain the unsymmetrical squaraine dyes as blue dyes.

#### 3.4.1. Synthesis of Unsymmetrical SQI-F Dye

A mixture of compound **3a** (1 g, 1.59 mmol, 1 equiv.) and compound **5** (0.5 g, 1.59 mmol, 1 equiv.) was dissolved in a 50 mL round-bottom flask in a mixture of n-butanol/toluene (20 mL, 1:1 *V*/*V*). The mixture was heated and refluxed under an inert atmosphere for 22 h using a Dean–Stark apparatus. After cooling to RT, the solvent was removed. The resultant crude product was purified by column chromatography (60–120 mesh) using a mixture of DCM/MeOH in a 10:0.1 (*V*/*V*) ratio to obtain SQI-F as a blue dye in a yield of 43%. IR (cm^−1^): 2955, 2918, 2849, 1699, 1598, 1567, 1505, 1497, 1466, 1444, 1357, 1263, 1204, 1174, 1084, 1050, 937, 842, 814, 795, 770, 735, 710, 685.^1^H NMR-CDCl_3_-400 MHz: δ0.49 (2H, CH_2_), 0.77 (2H, CH_2_), 0.86 (m, 8H, 4CH_2_), 1.3–1.1 (m, 52H, 26CH_2_), 1.4 (m, 2H, CH_2_), 1.8 (s, 9H), 1.99–1.96 (m, 2H, CH_2_), 3.0 (2H, CH_2_), 3.5 (s, 3H, CH_3_), 4.05 (2H, CH_2_), 5.98 (s, 1H, =CH-), 6.14 (s, 1H, =CH-),7.0 (d, 2H, Ar-H, *J* = 8 Hz), 7.27 (ds, 2H, Ar-H, *J* = 8 Hz), 8.1 (s, 1H, Ar-H), 8.13 (d, 1H, Ar-H, *J* = 8 Hz). ^13^CNMR-CDCl_3_: δ 14.14, 22.65, 22.71, 29.28, 29.36, 29.48, 29.58, 29.63, 29.71, 31.92, 48.26, 59.55, 76.76, 77.07, 77.28, 77.38, 88.12, 88.54, 108.08, 110.20, 110.45, 114.48, 114.72, 123.49, 123.96, 124.38, 131.15, 139.94, 141.80, 147.36, 159.40, 161.84, 168.82, 170.39, 170.74, 176.45, 181.68. HRMS (*m*/*z*): [M + H]^+^ calculated for C_60_H_88_FN_2_O_4_: 920.3724, found, 921.799.

#### 3.4.2. Synthesis of Unsymmetrical SQI-Cl Dye

A mixture of compound **3b** (1 g, 1.55 mmol, 1 equiv.) and compound **5** (0.5 g, 1.55 mmol, 1 equiv.) was dissolved in a 50 mL round-bottom flask in a mixture of n-butanol/toluene (20 mL, 1:1 *V*/*V*). The mixture was heated and refluxed under an inert atmosphere for 22 h using a Dean–Stark apparatus. After cooling to RT, the solvent was removed. The resultant crude product was purified by column chromatography (60–120 mesh) using a mixture of DCM/MeOH in a 10:0.1 (*V*/*V*) ratio to obtain SQI-F as a blue dye in a yield of 47%. IR (cm^−1^): 2954, 2919, 2848, 1697, 1598, 1568, 1504, 1487, 1464, 1429, 1354, 1260, 1205, 1175, 1084, 1053, 936, 840, 810, 785, 740, 685, 665.^1^H NMR (CDCl_3_-400 MHz): δ0.49 (2H, CH_2_), 0.76 (2H, CH_2_), 0.83–0.86 (m, 8H, 4CH_2_), 1.1–1.3 (m, 54H, 27CH_2_), 1.81 (m, 2H, CH_2_), 1.84 (t, 9H, 3CH_3_), 1.96–1.99 (m, 2H, CH_2_), 3.0 (2H, CH_2_), 3.5 (s, 3H, CH_3_), 4.02 (2H, CH_2_), 6.0 (s, 1H, =CH-), 6.15 (s, 1H, =CH-), 6.92–7.0 (dd, 2H, Ar-H, *J* = 8 Hz), 7.27–7.33 (ds, 2H, Ar-H, *J* = 8 Hz), 8.1 (s, 1H, Ar-H), 8.13 (d, 1H, Ar-H, *J* = 8 Hz). ^13^CNMR-CDCl_3_: δ 14.14, 22.72, 24.09, 29.29, 29.37, 29.49, 29.55, 29.64, 29.72, 31.92, 48.37, 59.32, 76.72, 77.04, 77.25, 77.36, 88.30, 108.24, 110.42, 122.83, 124.01, 124.01, 127.99, 130.07, 131.26, 141.91, 142.61, 147.47, 169.20, 170.01, 178.06, 182.67. HRMS (*m*/*z*): [M + H]^+^ calculated for C_60_H8_8_Cl N_2_O_4_: 936.824, found, 937.726.

### 3.5. DSSC Fabrication Procedure

The fabrication of DSSCs commenced with an ITO glass substrate that possessed a sheet resistance between 8–10 Ω/sq. It was thoroughly cleaned in a two-step process: initially with a 2% mucasol solution in de-ionized water (DI-H_2_O), followed by isopropanol, with both steps enhanced by ultrasonic agitation. The cleaned ITO substrate was treated in a 40 mM TiCl_4_ solution at 70 °C for 25 min. Post treatment, it was washed with DI-H_2_O and ethanol and dried at 110 °C for 15 min. A solution of TiO_2_ nanoparticles, with sizes under 20nm, was coated on the conductive face of the ITO substrate using the doctor-blade technique. This layer was allowed to air-dry for 15 min before being thermally treated at 120 °C for 30 min. Subsequently, a TiO_2_ paste scattering layer was applied. This was then thermally treated at 120 °C for another 15 min. A systematic sintering of the substrate followed, where it was exposed to increasing temperatures: 350 °C, 400 °C, 450 °C, and finally, 500 °C. Each temperature was maintained for 15 min, escalating at a rate of 5 °C per min. After sintering, the substrate was treated again with the 40 mM TiCl_4_ solution at 70 °C for 30 min, washed, and sintered at 500 °C for a final 30 min. The resultant substrate, with a TiO_2_ surface area of 0.23 cm^2^, was immersed in a solution containing the SQI-F and SQI-Cl dyes. This immersion lasted for 15 h in darkness. The assembly of the cell incorporated a platinum foil counter electrode and a 25 μm spacer. The electrolyte used was a combination of iodolyte Z-50 and Z-100, sourced from Solaronix. The active area of the cell was 0.36 cm^2^, defined by a black mask. The performance of the dyes, SQI-F and SQI-Cl, was tested under standard solar simulator settings (AM 1.5 G at 100 mW/cm^2^).

## 4. Conclusions

In an effort to harness the potential of halogen bonding for enhancing the *J_SC_* and efficiency of DSSCs, we synthesized and characterized SQI-F and SQI-Cl dyes. These dyes are based on halogen atom-functionalized D-A-D unsymmetrical squaraine structures, specifically designed for DSSC applications. Devices fabricated using these dyes, in conjunction with iodolyte electrolytes (Z-50 and Z-100), were further optimized with CDCA. Both SQI-F and SQI-Cl dyes displayed various photovoltaic characteristics with both electrolytes when CDCA was not used. However, when sensitized with (3 equiv.) of CDCA, these dyes exhibited improved photovoltaic performance compared to their non-CDCA sensitized counterparts. Notably, the SQI-F dye achieved the highest DSSC device efficiency of 6.74%, with a *V_OC_* of 0.694 V and a *J_SC_* of 13.67 mA/cm^2^ when paired with the iodolyte Z-100 electrolyte and sensitized with (3 equiv.) of CDCA. The theoretical calculations and absorbance results indicate that the electron density in the LUMO of SQI-F and SQI-Cl is distributed across the entire carboxylic group, leading to a robust electronic interaction between the dye sensitizers and the conduction band of TiO_2_. This enhanced efficiency and *J_SC_* in the Z-100 electrolyte can be attributed to the presence of a σ-hole, which strengthens the interaction between the electrolyte and the dyes on the TiO_2_ surface, thereby facilitating the dye regeneration process.

## Figures and Tables

**Table 1:** Photophysical and electrochemical attributes of SQI-F and SQI-Cl.

Dye	*λ*_maxa_ (nm) ^a^	Ɛ_max_ (M^−1^ cm^−1^) ^a^	*λ*_em_ (nm) ^a^	*λ*_max_ TiO_2_ (nm)	LHE Δ*λ* (60%) (nm)	E_oxd_^c^ (V vs. NHE)	E_g_ (eV) ^b^	HOMO (eV) ^c^	LUMO (eV) ^c^	HOMO (eV) ^d^	LUMO (eV) ^d^	E_g_ (eV) ^d^
SQI-F	641	2.1 × 10^5^	643	642	195	0.81	1.92	−6.01	−4.09	−5.14	−3.13	2.01
SQI-Cl	643	2.5 × 10^5^	653	651	213	0.83	1.93	−6.03	−4.1	−5.16	−3.17	1.99

^a^ Measured in EOH solution. ^b^ The determination of the band gap was accomplished by analyzing emission and absorption spectra. ^c^ The oxidation potential (E_ox_) was assessed in a solution of 0.1 M TBAPF_6_ in DMSO, employing a three-electrode configuration comprising a platinum counter electrode, a reference electrode of silver/silver chloride, and a working electrode of platinum. Calibration was executed utilizing the ferrocene/ferrocenium (Fc/Fc^+^) redox pair. ^d^ Computational calculations were executed utilizing the Gaussian 09 software suite.

**Table 2:** A comparative analysis of the photo-electrochemical characteristics of SQI-F and SQI-Cl, both with and without CDCA, in ^a^ Z-50 and ^a^ Z-100 electrolytes with ^b^ SQIND and ^c^ SQ-A.

Dyes	CDCA (mM)	*Jsc* (mA/cm^2^) Z-50/Z-100	*Voc* (V) Z-50/Z-100	FF Z-50/Z-100	η (%) Z-50/Z-100
SQI-F	0	10.70/12.4	0.720/0.693	0.75/0.73	5.62/6.01
SQI-Cl	0	11.12/12.22	0.704/0.683	0.72/0.72	5.47/5.84
SQI-F	0.3	12.22/13.67	0.723/0.694	0.75/0.73	6.46/6.74
SQI-Cl	0.3	11.20/12.09	0.707/0.680	0.72/0.71	5.55/5.64
^b^ SQIND-1	0.3	5.88	0.48	0.74	2.1
^c^ SQ-A	0	6.71	0.58	0.72	2.82

^a^ The concentrations of I_3_^−^ were set at 50 mM and 100 mM. ^b^ SQIND-1 [42]. ^c^ SQ-A [28].

**Table 3:** EIS parameters for SQI-F and SQI-Cl dyes under dark conditions using iodolyte electrolytes (^a^ Z-50 and ^a^ Z-100).

Dye	R_ct_ (Ω cm^2^) Z-50/Z-100	*C*_μ_ (mF) Z-50/Z-100	τ (ms) Z-50/Z-100
SQI-F	4.42/4.45	0.394/0.43	1.74/1.83
SQI-F (CDCA 1:3)	4.87/4.91	0.415/0.42	2.02/1.96
SQI-Cl	4.67/6.03	0.434/0.439	2.02/2.64
SQI-Cl (CDCA 1:3)	4.64/5.52	0.430/0.403	2.64/2.22

^a^ The concentrations of I_3_^−^ were set at 50 mM and 100 mM.

## Data Availability

The data supporting the findings of this study can be obtained from the corresponding author upon reasonable request.

## References

[1] Al-horaibi S.A., Al-Odayni A.-B., Alezzy A., ALSaeedy M., Al-Adhreai A., Saeed W.S., Hasan A. (2023). Novel Squaraine Dyes for High-Performance in Dye-Sensitized Solar Cells: Photophysical Properties and Adsorption Behavior on TiO_2_ with Different Anchoring Groups. J. Mol. Struct..

[2] Al-horaibi S.A., Al-Odayni A.-B., Alezzy A., ALSaeedy M., Saeed W., Hasan A., El-Shishtawy R.M. (2023). Development of New Co-Sensitizer Based Squaraine Dyes for Enhancing the Performance of DSSC. J. Mol. Struct..

[3] Mishra A., Fischer M.K.R., Bäuerle P. (2009). Metal-Free Organic Dyes for Dye-Sensitized Solar Cells: From Structure: Property Relationships to Design Rules. Angew. Chem. Int. Ed..

[4] Saygili Y., Stojanovic M., Flores-Díaz N., Zakeeruddin S.M., Vlachopoulos N., Grätzel M., Hagfeldt A. (2019). Metal Coordination Complexes as Redox Mediators in Regenerative Dye-Sensitized Solar Cells. Inorganics.

[5] Wu Y., Zhu W. (2013). Organic Sensitizers from D–π–A to D–A–π–A: Effect of the Internal Electron-Withdrawing Units on Molecular Absorption, Energy Levels and Photovoltaic Performances. Chem. Soc. Rev..

[6] Mughal E.U., Obaid R.J., Sadiq A., Alsharif M.A., Naeem N., Kausar S., Altaf A.A., Jassas R.S., Ahmed S., Alsantali R.I. (2022). Chalcone-and Flavone-Based Novel Terpyridine Metal Complexes: Synthesis, Electrochemical, Photophysical, Photovoltaic and Computational Studies. Dyes Pigments.

[7] Qin C., Numata Y., Zhang S., Yang X., Islam A., Zhang K., Chen H., Han L. (2014). Novel Near-Infrared Squaraine Sensitizers for Stable and Efficient Dye-Sensitized Solar Cells. Adv. Funct. Mater..

[8] Yang L., Zheng Z., Li Y., Wu W., Tian H., Wang Z. (2015). N-Annulated Perylene-Based Metal-Free Organic Sensitizers for Dye-Sensitized Solar Cells. Chem. Commun..

[9] Qin C., Islam A., Han L. (2012). Incorporating a Stable Fluorenone Unit into D–A–π–A Organic Dyes for Dye-Sensitized Solar Cells. J. Mater. Chem..

[10] Zhong C., Gao J., Cui Y., Li T., Han L. (2015). Coumarin-Bearing Triarylamine Sensitizers with High Molar Extinction Coefficient for Dye-Sensitized Solar Cells. J. Power Sources.

[11] Abusaif M.S., Fathy M., Abu-Saied M.A., Elhenawy A.A., Kashyout A.B., Selim M.R., Ammar Y.A. (2021). New Carbazole-Based Organic Dyes with Different Acceptors for Dye-Sensitized Solar Cells: Synthesis, Characterization, Dssc Fabrications and Density Functional Theory Studies. J. Mol. Struct..

[12] Daeneke T., Mozer A.J., Uemura Y., Makuta S., Fekete M., Tachibana Y., Koumura N., Bach U., Spiccia L. (2012). Dye Regeneration Kinetics in Dye-Sensitized Solar Cells. J. Am. Chem. Soc..

[13] Singh A.K., Nithyanandhan J. (2021). Amphiphilic Indoline-Based Unsymmetrical Squaraine Dyes for Dye-Sensitized Solar Cells: Modulating the Dye-TiO_2_/Electrolyte Interface for Nonaqueous and Aqueous Electrolytes. ACS Appl. Energy Mater..

[14] Singh A.K., Mele Kavungathodi M.F., Nithyanandhan J. (2019). Alkyl-Group-Wrapped Unsymmetrical Squaraine Dyes for Dye-Sensitized Solar Cells: Branched Alkyl Chains Modulate the Aggregation of Dyes and Charge Recombination Processes. ACS Appl. Mater. Interfaces.

[15] Alagumalai A., Vellimalai P., Sil M.C., Nithyanandhan J. (2016). Effect of Out-of-Plane Alkyl Group’s Position in Dye-Sensitized Solar Cell Efficiency: A Structure--Property Relationship Utilizing Indoline-Based Unsymmetrical Squaraine Dyes. ACS Appl. Mater. Interfaces.

[16] Anderson A.Y., Barnes P.R.F., Durrant J.R., O’Regan B.C. (2011). Quantifying Regeneration in Dye-Sensitized Solar Cells. J. Phys. Chem. C.

[17] Otsuka A., Funabiki K., Sugiyama N., Yoshida T., Minoura H., Matsui M. (2006). Dye Sensitization of ZnO by Unsymmetrical Squaraine Dyes Suppressing Aggregation. Chem. Lett..

[18] Parlane F.G.L., Mustoe C., Kellett C.W., Simon S.J., Swords W.B., Meyer G.J., Kennepohl P., Berlinguette C.P. (2017). Spectroscopic Detection of Halogen Bonding Resolves Dye Regeneration in the Dye-Sensitized Solar Cell. Nat. Commun..

[19] Simon S.J.C., Parlane F.G.L., Swords W.B., Kellett C.W., Du C., Lam B., Dean R.K., Hu K., Meyer G.J., Berlinguette C.P. (2016). Halogen Bonding Promotes Higher Dye-Sensitized Solar Cell Photovoltages. J. Am. Chem. Soc..

[20] Curiac C., Hunt L.A., Sabuj M.A., Li Q., Baumann A., Cheema H., Zhang Y., Rai N., Hammer N.I., Delcamp J.H. (2021). Probing Interfacial Halogen-Bonding Effects with Halogenated Organic Dyes and a Lewis Base-Decorated Transition Metal-Based Redox Shuttle at a Metal Oxide Interface in Dye-Sensitized Solar Cells. J. Phys. Chem. C.

[21] Malzner F.J., Brauchli S.Y., Constable E.C., Housecroft C.E., Neuburger M. (2014). Halos Show the Path to Perfection: Peripheral Iodo-Substituents Improve the Efficiencies of Bis (Diimine) Copper (I) Dyes in DSCs. RSC Adv..

[22] Swords W.B., Simon S.J.C., Parlane F.G.L., Dean R.K., Kellett C.W., Hu K., Meyer G.J., Berlinguette C.P. (2016). Evidence for Interfacial Halogen Bonding. Angew. Chem..

[23] Cavallo G., Metrangolo P., Milani R., Pilati T., Priimagi A., Resnati G., Terraneo G. (2016). The Halogen Bond. Chem. Rev..

[24] Kellett C.W., Kennepohl P., Berlinguette C.P. (2020). π Covalency in the Halogen Bond. Nat. Commun..

[25] Raithel A.L., Meador W.E., Kim T.-Y., Staples R.J., Delcamp J.H., Hamann T.W. (2023). Molecular Switch Cobalt Redox Shuttle with a Tunable Hexadentate Ligand. J. Am. Chem. Soc..

[26] Ji J.-M., Lee H.J., Zhou H., Eom Y.K., Kim C.H., Kim H.K. (2022). Influence of the π-Bridge-Fused Ring and Acceptor Unit Extension in D− π–A-Structured Organic Dyes for Highly Efficient Dye-Sensitized Solar Cells. ACS Appl. Mater. Interfaces.

[27] Nugegoda D., Hunt L.A., Devdass A., Cheema H., Fortenberry R.C., Jurss J.W., Hammer N.I., Delcamp J.H. (2022). Lewis Acid–Lewis Base Interactions Promote Fast Interfacial Electron Transfers with a Pyridine-Based Donor Dye in Dye-Sensitized Solar Cells. ACS Appl. Energy Mater..

[28] Shivashimpi G.M., Pandey S.S., Watanabe R., Fujikawa N., Ogomi Y., Yamaguchi Y., Hayase S. (2014). Effect of Nature of Anchoring Groups on Photosensitization Behavior in Unsymmetrical Squaraine Dyes. J. Photochem. Photobiol. A Chem..

[29] Bisht R., Sudhakar V., Mele Kavungathodi M.F., Karjule N., Nithyanandhan J. (2018). Fused Fluorenylindolenine-Donor-Based Unsymmetrical Squaraine Dyes for Dye-Sensitized Solar Cells. ACS Appl. Mater. Interfaces.

[30] Punitharasu V., Mele Kavungathodi M.F., Singh A.K., Nithyanandhan J. (2019). $π$-Extended Cis-Configured Unsymmetrical Squaraine Dyes for Dye-Sensitized Solar Cells: Panchromatic Response. ACS Appl. Energy Mater..

[31] Watson J., Rodrigues R.R., Delcamp J.H. (2022). Near-Infrared Unsymmetrical Squaraine Core-Based Sensitizers for Co-Sensitized High-Photocurrent Dye-Sensitized Solar Cells. Cell Rep. Phys. Sci..

[32] Islam N., Niaz S., Manzoor T., Pandith A.H. (2014). Theoretical Investigations into Spectral and Non-Linear Optical Properties of Brucine and Strychnine Using Density Functional Theory. Spectrochim. Acta Part A Mol. Biomol. Spectrosc..

[33] Ben Messaoudaa M., Slimic H., Abderrabbaa M., ben Salemc R., Moussaouic Y. (2017). The Biginelli Reaction in Different Solvents and in Presence of Bismuth Nitrate: Thermodynamical Investigation into the Mechanism by Means of DFT Calculation and Experimental Results. J. Tunis. Chem. Soc..

[34] Singh G., Singh R., George N., Singh G., Kaur G., Kaur G., Singh H., Singh J. (2023). ‘Click’-Synthesized PET Based Fluorescent Sensor for Hg (II), Pb (II) and Cr (III) Recognition: DFT and Docking Studies. J. Photochem. Photobiol. A Chem..

[35] Singh A.K., Kavungathodi M.F.M., Mozer A.J., Krishnamoorthy K., Nithyanandhan J. (2022). Solvent-Dependent Functional Aggregates of Unsymmetrical Squaraine Dyes on TiO_2_ Surface for Dye-Sensitized Solar Cells. Langmuir.

[36] Pradhan A., Morimoto T., Saikiran M., Kapil G., Hayase S., Pandey S.S. (2017). Investigation of the Minimum Driving Force for Dye Regeneration Utilizing Model Squaraine Dyes for Dye-Sensitized Solar Cells. J. Mater. Chem. A.

[37] Jradi F.M., Kang X., Oneil D., Pajares G., Getmanenko Y.A., Szymanski P., Parker T.C., El-Sayed M.A., Marder S.R. (2015). Near-Infrared Asymmetrical Squaraine Sensitizers for Highly Efficient Dye Sensitized Solar Cells: The Effect of π-Bridges and Anchoring Groups on Solar Cell Performance. Chem. Mater..

[38] Bisht R., Singh A.K., Nithyanandhan J. (2017). Panchromatic Sensitizer for Dye-Sensitized Solar Cells: Unsymmetrical Squaraine Dyes Incorporating Benzodithiophene $π$-Spacer with Alkyl Chains to Extend Conjugation, Control the Dye Assembly on TiO_2_, and Retard Charge Recombination. J. Org. Chem..

[39] Matsui M., Haishima Y., Kubota Y., Funabiki K., Jin J., Kim T.H., Manseki K. (2017). Application of Benz [c, d] Indolenine-Based Unsymmetrical Squaraine Dyes to near-Infrared Dye-Sensitized Solar Cells. Dyes Pigments.

[40] Pradhan S., Kurokawa Y., Pandey S.S. (2023). Design and Synthesis of Novel NIR-Sensitive Unsymmetrical Squaraine Dyes for Molecular Photovoltaics. Phys. Status Solidi.

[41] Singh A.K., Maibam A., Javaregowda B.H., Bisht R., Kudlu A., Krishnamurty S., Krishnamoorthy K., Nithyanandhan J. (2020). Unsymmetrical Squaraine Dyes for Dye-Sensitized Solar Cells: Position of the Anchoring Group Controls the Orientation and Self-Assembly of Sensitizers on the TiO_2_ Surface and Modulates Its Flat Band Potential. J. Phys. Chem. C.

[42] Al-horaibi S.A., Asiri A.M., El-Shishtawy R.M., Gaikwad S.T., Rajbhoj A.S. (2019). Synthesis and Characterization of New Squaraine Dyes with Bis-Pendent Carboxylic Groups for Dye-Sensitized Solar Cells. J. Mol. Struct..

[43] Al-horaibi S.A., Garoon E.M., Bhise N.A., Gaikwad S.T., Rajbhoj A.S. (2020). The Effect of Bis-Carboxylic Groups of Squarylium Dyes on the Efficiency of Dye-Sensitized Solar Cells. Chem. Pap..

[44] Shivashimpi G.M., Pandey S.S., Watanabe R., Fujikawa N., Ogomi Y., Yamaguchi Y., Hayase S. (2012). Novel Unsymmetrical Squaraine Dye Bearing Cyanoacrylic Acid Anchoring Group and Its Photosensitization Behavior. Tetrahedron Lett..

[45] Al-Horaibi S.A., Alghamdi M.T., Gaikwad S.T., Rajbhoj A.S. (2018). Comparison and Determine Characteristics Potentials of HOMO/LUMO and Relationship between Ea and Ip for Squaraine Dyes (SQ1, SQ2) by Using Cyclic Voltammetry and DFT/TD-DFT. Moroc. J. Chem..

[46] Al-Sehemi A.G., Irfan A., Al-Melfi M.A.M., Al-Ghamdi A.A., Shalaan E. (2014). The Enhanced Efficiency to 3.6% Based on Organic Dye as Donor and Si/TiO_2_ Acceptor Bulk Hetero-Junction Solar Cells. J. Photochem. Photobiol. A Chem..

[47] Al-Sehemi A.G., Irfan A., Asiri A.M., Ammar Y.A. (2012). Molecular Design of New Hydrazone Dyes for Dye-Sensitized Solar Cells: Synthesis, Characterization and DFT Study. J. Mol. Struct..

